# Multimodal large language models for oral lesion diagnosis: a systematic review of diagnostic performance and clinical utility

**DOI:** 10.3389/froh.2026.1748450

**Published:** 2026-02-24

**Authors:** Fatma E. A. Hassanein, Malik Alkabazi, Melek Tassoker, Yousra Ahmed, Suliman Alsaeed, Asmaa Abou-Bakr

**Affiliations:** 1 Department of Oral Medicine, Periodontology, and Oral Diagnosis, Faculty of Dentistry, King Salman International University, El Tur, South Sinai, Egypt; 2 Faculty of Dentistry Khalij-Libya, Tripoli, Libya; 3 Department of Dentomaxillofacial Radiology, Faculty of Dentistry, Necmettin Erbakan University, Meram, Konya, Türkiye; 4 Department of Prosthetic Dentistry, Removable Prosthodontic Division, Faculty of Dentistry, King Salman International University, El Tur, South Sinai, Egypt; 5 Preventive Dental Sciences Department, College of Dentistry, King Saud Bin Abdulaziz University for Health Sciences, Riyadh, Saudi Arabia; 6 King Abdullah International Medical Research Center, Riyadh, Saudi Arabia; 7 Ministry of the National Guard—Health Affairs, Riyadh, Saudi Arabia; 8 Department of Oral Medicine and Periodontology, Faculty of Dentistry, Galala University, Suez, Egypt

**Keywords:** artificial intelligence in dentistry, clinical decision support, diagnostic accuracy, large language models, multimodal AI, oral lesions

## Abstract

**Background:**

Diagnosing oral lesions from benign conditions to oral cancer remains challenging due to overlapping visual features and reliance on histopathology. Large language models (LLMs) can integrate textual and visual cues, but their diagnostic accuracy and clinical utility in real decision-making contexts remain uncertain. To systematically evaluate the diagnostic performance, clinical usefulness, and limitations of LLMs in identifying oral lesions.

**Methods:**

PubMed, CINAHL, Embase, Web of Science, and Google Scholar were searched to 20 July 2025. Eligible studies applied LLMs (e.g., ChatGPT, Gemini, DeepSeek, Copilot, Claude) for diagnosis or differential diagnosis of oral lesions using text, images, or multimodal inputs. Outcomes included diagnostic accuracy, agreement metrics, and qualitative assessments of explanation quality and clinical applicability. Risk of bias was assessed using an adapted QUADAS-2. Narrative synthesis was performed due to heterogeneity.

**Results:**

Seventeen studies (>1,200 cases) were included. Diagnostic accuracy ranged from 25%–96%, varying by model version, input modality, and lesion complexity. Multimodal inputs consistently improved performance, with Cohen's κ up to 0.85–0.90. Advanced models (GPT-4o, DeepSeek-R1, o1-preview) outperformed earlier versions and approached expert performance in some tasks, although specialists generally retained superior Top-1 accuracy. Clinical utility was highest when LLMs were used to structure differential reasoning, highlight red-flag features, and support communication, but limited in tasks requiring fine morphological interpretation or severity grading. Overall risk of bias was low to moderate.

**Conclusions:**

LLMs demonstrate variable diagnostic performance and context-dependent supportive utility as adjunctive tools in oral lesion assessment, particularly in multimodal settings. They should complement, rather than replace, expert clinical judgment. Future research should prioritize real-world workflow evaluation, standardized prompting strategies, and prospective clinical validation.

**Systematic Review Registration:**

https://www.crd.york.ac.uk/PROSPERO/view/CRD420251090315, identifier CRD420251090315.

## Introduction

In clinical practice, prompt and precise diagnosis of oral lesions, which can range from benign conditions to potentially malignant disorders (PMDs) and oral squamous cell carcinoma (OSCC), remains a major challenge. Even for skilled medical professionals, the visual resemblance of numerous benign, dysplastic, and malignant lesions frequently causes diagnostic ambiguity (1, 2). This is crucial because delayed diagnosis of OSCC is associated with advanced disease stage, complex treatment pathways, and persistently low five-year survival rates (3, 4).

Histopathological analysis of a tissue biopsy, the current reference standard for conclusive diagnosis, is invasive, time-consuming, and resource-dependent. Therefore, there is an urgent need for accurate, rapid, and accessible decision-support tools to assist initial assessment and triage, particularly in primary care settings where early presentations commonly occur.

The field of medical diagnostics is undergoing rapid evolution with artificial intelligence (AI), particularly deep learning. Convolutional neural networks (CNNs) have demonstrated expert-level image interpretation in dentistry, showing strong performance in detecting periapical lesions, periodontal disease, and caries (5, 6). AI models have also achieved high sensitivity and specificity in distinguishing benign from malignant oral lesions using clinical photographs (2, 7–10). Prior systematic reviews have established a strong foundation for AI-assisted diagnostic support across dental specialties (11, 12).

However, large language models (LLMs) such as GPT-4 (OpenAI), Gemini (Google), and LLaMA (Meta) represent a distinct paradigm shift. Their advanced natural language processing capabilities allow them to synthesize clinical histories, describe reasoning steps, and generate explanations, rather than simply classify images. Multimodal LLMs can now integrate text and visual data, including clinical descriptions, photographic images, and even histopathology reports (13).

This positions LLMs as potential clinical reasoning tools rather than mere image classifiers. An LLM can take combined inputs (patient history + lesion image + clinical descriptors) to produce differential diagnoses, justify its reasoning, identify red-flag features, and suggest next steps in management (14). This aligns directly with real-world oral medicine workflows, where diagnostic accuracy depends on integrating multiple information sources.

Although promising, the diagnostic use of LLMs for oral lesions remains nascent, and existing evidence is fragmented. Previous reviews have focused either on histology-based machine learning for head and neck cancer (12) or general applications of AI in dentistry (11, 15). A gap remains regarding the diagnostic accuracy, clinical utility, and limitations of LLMs specifically in oral mucosal disease, including performance across text-only, image-only, and multimodal workflows, comparison to human expertise, and susceptibility to bias.

This systematic review was therefore designed to evaluate the diagnostic performance and clinical utility of LLMs in identifying oral lesions, while also examining their reported limitations and risks of bias. In contrast to prior broad AI reviews, this work adopts a focused scope, emphasizing the unique reasoning capabilities and multimodal diagnostic potential of LLMs.

## Methods

### Protocol and registration

This review was prospectively registered in PROSPERO (CRD420251090315) on 09/07/2025 and conducted in accordance with the registered protocol. No deviations from the protocol occurred.

### Eligibility criteria

We defined eligibility criteria *a priori* in line with PRISMA 2020 recommendations.

#### Inclusion criteria

Studies applying LLMs (e.g., ChatGPT, Gemini, DeepSeek, Copilot, Claude, LLaMA) to generate a final diagnosis or differential diagnosis for oral mucosal or jaw lesions (benign, potentially malignant, or malignant) were included. Accepted input modalities encompassed text-only, image-only, and multimodal combinations (clinical descriptions, histopathology reports, photographic images). Outcomes included diagnostic accuracy or expert agreement. Both real patient cases and standardized vignette-based evaluations were eligible when the diagnostic task reflected real decision-making.

#### Exclusion criteria

Studies were excluded if they: (1) relied solely on conventional machine learning/CNN-based imaging models without an LLM; (2) did not involve diagnostic inference (e.g., education-only applications); or (3) were review articles, commentaries, or non–patient-derived reports.

Eligibility is summarized in Table 1 (PICOS framework).

**Table 1:** Eligibility criteria based on PICOS framework.

Domain	Specification
Population (P)	Patients with oral mucosal or jaw lesions (benign, premalignant, or malignant), are described through clinical, histological, photographic, or multimodal datasets.
Intervention (I)	Application of large language models (LLMs) chatbots (e.g., GPT, Gemini, DeepSeek, LLaMA, copilot, Claude) for generating diagnostic or differential diagnosis outputs.
Comparator (C)	Human expert diagnosis (oral medicine specialists, pathologists, or dentists) and/or reference standards (e.g., histopathology, established diagnostic criteria). Studies without comparators were eligible if diagnostic outcomes were reported.
Outcomes (O)	Primary: Objective diagnostic performance and reliability metrics, including accuracy, sensitivity, specificity, PPV, NPV, AUC, Top-k accuracy, and agreement coefficients (e.g., Cohen's κ, Gwet's AC1), reflecting diagnostic validity relative to the reference standard.
	Secondary: Subjective and perception-based measures of model output quality and usability, including explanation quality, plausibility ratings, interpretability, narrative usefulness, and perceived clinical utility, which do not represent diagnostic correctness.
Study Design (S)	Diagnostic accuracy studies (prospective or retrospective), vignette-based experimental evaluations, and multimodal validation studies.
	Excluded: reviews, editorials, commentaries, surveys of educational settings, or studies outside lesion diagnosis.

### Outcomes

Outcomes were predefined and categorized into two distinct domains. Primary outcomes included objective diagnostic performance and reliability measures, such as accuracy, sensitivity, specificity, positive and negative predictive values, area under the receiver operating characteristic curve (AUC), Top-k accuracy, and agreement coefficients (e.g., Cohen's κ and Gwet's AC1), reflecting diagnostic validity relative to the applied reference standard.

Secondary outcomes comprised subjective and perception-based measures of model output quality and usability, including explanation quality, plausibility ratings, interpretability, narrative usefulness, and perceived clinical utility. These outcomes represent qualitative assessments of reasoning support and user experience and do not constitute measures of diagnostic correctness.

Primary and secondary outcome domains were analyzed and reported separately to avoid conceptual overlap and overinterpretation of clinical relevance.

### Search strategy

A comprehensive search was performed in PubMed, CINAHL, Embase, Web of Science, and ScienceDirect covering the period from January 2022 to 20 July 2025, using MeSH terms and free-text keywords related to large language models and oral lesions. No date limits were applied. English-language restriction was applied due to the limited comparability of cross-language LLM performance. Full search strategies are provided in Supplementary Appendix 1 (PRISMA-S).

### Study selection

Records were deduplicated in EndNote and screened independently by two reviewers in Rayyan. Discrepancies were resolved by a third reviewer. Inter-reviewer agreement was calculated using Cohen's κ for both title/abstract and full-text stages. The single included study with overlapping authorship was evaluated exclusively by reviewers without overlapping authorship, with discrepancies resolved by an independent third reviewer.

### Data extraction and management

Data extraction was conducted independently using a standardized, piloted form and verified by a third reviewer. Extracted items included study design, sample characteristics, model version, input modality, comparator, lesion type, and outcomes (accuracy, sensitivity, specificity, AUC, agreement coefficients, explanation quality).

### Risk of bias assessment

Risk of bias was appraised using an adapted QUADAS-2 tailored for LLM-based diagnostic evaluations, assessing case selection, index test, reference standard, and flow/timing. Judgments were categorized as low, high, or some concerns. Summary and traffic-light visualizations were generated (Supplementary Appendix 2).

### Data synthesis

Due to anticipated clinical and methodological heterogeneity across large language model families, input modalities (text-only, image-only, and multimodal), lesion domains (oral mucosal and jaw lesions), comparator reference standards, and outcome definitions, meta-analysis was not undertaken. Instead, a structured narrative synthesis was performed. Findings were organized according to: (1) LLM family, (2) input modality, (3) comparator reference standard, and (4) lesion category. Descriptive statistics were used to summarize, as distinct outcome domains, study-level diagnostic performance metrics, agreement/reliability measures, and utility-related or subjective outcomes as reported in the original studies. No pooled estimates (e.g., means or summary effect sizes) were calculated; results were presented descriptively using individual study values and ranges, as appropriate, to avoid inappropriate aggregation across heterogeneous study designs.

## Results

### Study selection

**Figure 1:**
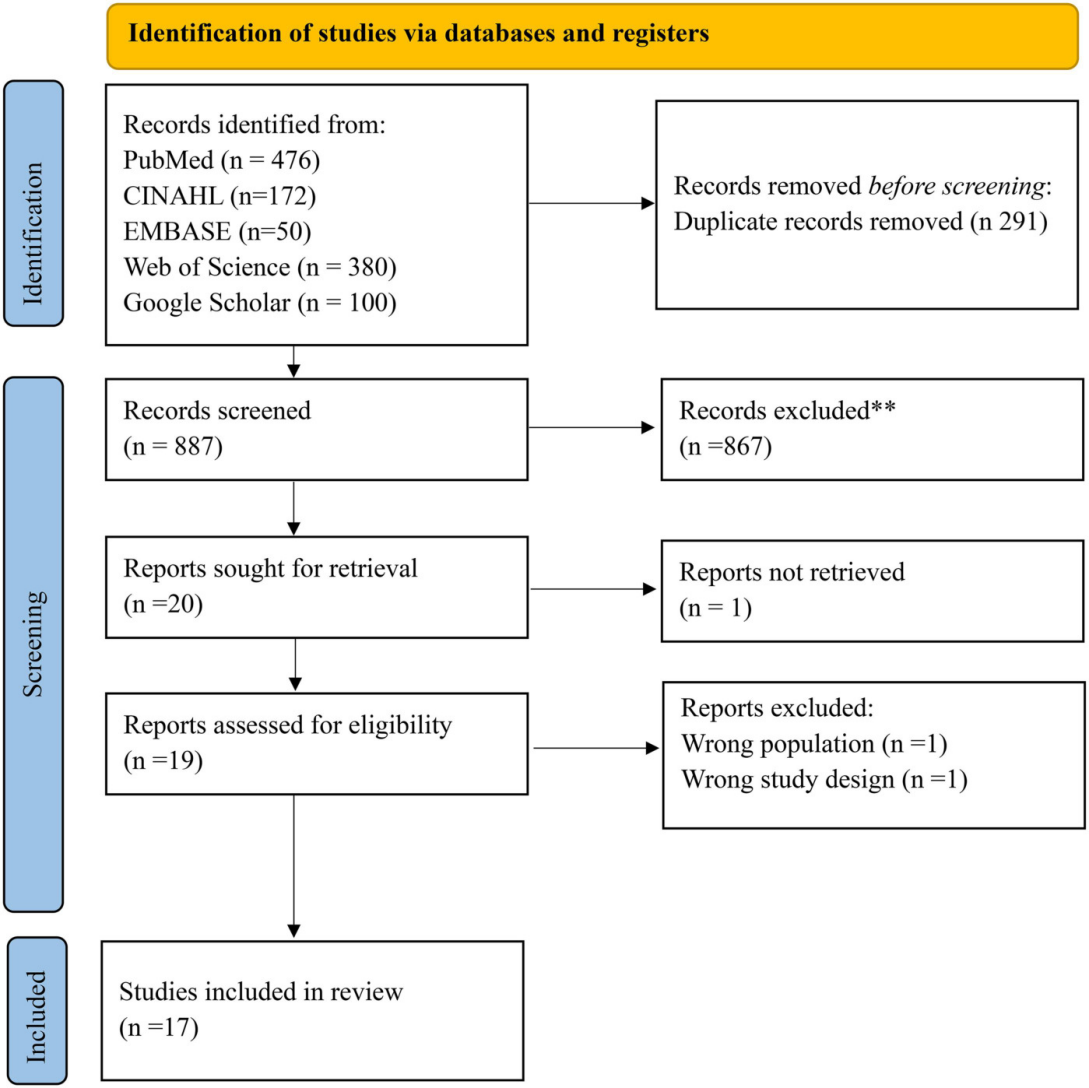
PRISMA chart.

The search identified 1,178 records (PubMed 476; CINAHL 172; EMBASE 50; Web of Science 380; Google Scholar 100). After removing 291 duplicates, 887 records were screened; 867 were excluded. 20 full texts were sought; 1 was not retrieved and 2 were excluded (wrong setting/population). 17 studies were included for qualitative synthesis (Figure 1).

Inter-reviewer agreement was substantial, with a Cohen's κ of 0.82 for title and abstract screening and 0.88 for full-text eligibility assessment.

### Study characteristics

The studies (published 2024–2025) evaluated a range of large language models (LLMs), including ChatGPT-3.5/4/4o/4 V, o1-preview, DeepSeek-V3/R1, Gemini 1.5 Pro/Flash, Claude 3/3.5, LLaMA 3.2, and Copilot (Table 2).

**Table 2:** Characteristics of included studies (*n* = 17).

Study	Model(s)	Clinical Domain	Modality	*n*	Reference Standard
Silva et al. (16)	ChatGPT-3.5	Radiolucent jaw lesions (DMFR)	Text	28	Histopathology
Schmidl et al. (24)	ChatGPT-4	OSCC/Leukoplakia/Benign oral lesions	Image; Text; Multimodal	45	Histopathology
Rewthamrongsris et al. (27)	GPT-4o, Gemini, Claude, LLaMA	OLP vs. non-OLP	Image	1,142	Histopathology
Pradhan (20)	ChatGPT-3.5/4/4o; Gemini	OPMLs & OSCC	Text; Multimodal	42	Expert consensus
Maia-Lima et al. (21)	GPT-4 transformer	Syndromic orofacial presentations	Multimodal	26	Expert consensus
Kaygisiz et al. (31)	GPT-4; DeepSeek-V3	Benign vs. malignant oral lesions	Text	16	Expert consensus
Yu et al. (28)	GPT-4o; Claude; Chat-Diagrams	OLP	Image	128	Histopathology
Tomo et al. (18)	ChatGPT-3.5/4; Human clinicians	11 oral lesion types	Text	37	Composite clinical criteria
Tassoker (17)	ChatGPT-4o; Experts	Mixed oral mucosal lesions	Multimodal	123	Expert consensus
Suárez et al. (22)	ChatGPT-4o	Oral/labial mucosal lesions	Image	30	Expert consensus
Hassanein et al. (25)	ChatGPT-4o; DeepSeek-V3; Experts	Mixed oral mucosal lesions	Multimodal	80	Histopathology
Diniz-Freitas et al. (23)	ChatGPT-4V	Oral diseases (NEJM + Oral Dis.)	Image; Text; Multimodal	57 (36 + 21)	Published case solutions
Diniz-Freitas et al. (19)	GPT-4o; DeepSeek-R1	Oral diseases (benchmarks)	Text	36	Published case solutions
Danesh et al. (29)	ChatGPT-3.5/4; o1-preview	Mixed dental/oral cases	Text	50	Published case solutions
Danesh et al. (38)	ChatGPT-3.5/4	Mixed dental/oral cases	Text	50	Published case solutions
Cuevas-Nuñez et al. (30)	ChatGPT-4	OMFP cases	Text	102	Expert consensus
AlFarabi Ali et al. (26)	ChatGPT-4; Copilot; Experts	Oral medicine clinical scenarios	Text	50	Histopathology + expert decision

OLP, oral lichen planus; OSCC, oral squamous cell carcinoma; OPMLs, oral potentially malignant lesions; OMFP, oral & maxillofacial pathology; Multi, multimodal. Reference standards: Histopathology 35% (6/17), Expert consensus 35% (6/17), Published cases 24% (4/17).

The included studies (2024–2025) evaluated a range of large language models, including ChatGPT-3.5/4/4o/4V, o1-preview, DeepSeek-V3/R1, Gemini 1.5 Pro/Flash, Claude 3/3.5, LLaMA 3.2, and Microsoft Copilot. Input modalities varied, with text-only approaches in 11 studies, image-only in 6 studies, and multimodal configurations in 6 studies, with some evaluating more than one modality. Lesion focus ranged from single-entity cohorts, such as OLP, OPMLs, leukoplakia, and syndromic lesions, to broader mixed oral mucosal case sets. Reference standards included histopathology/biopsy in four studies, expert consensus in seven studies, published clinical solutions in four studies, and hybrid standards in two studies. Sample sizes ranged from 16 to 1,142 cases (Table 2).

### Diagnostic performance

Comparative performance statements reported in this section are restricted to within-study evaluations; cross-study differences are presented descriptively and should not be interpreted as direct model rankings. Table 3 organizes outcomes into two distinct domains: diagnostic performance metrics (objective measures of accuracy and agreement) and subjective perception-based outcomes (plausibility and reasoning quality assessments). This separation prevents conceptual overlap and reduces the risk of overinterpretation of clinical relevance. Objective diagnostic performance varied substantially according to model family, input modality, lesion complexity, and study design (Table 3).

**Table 3:** Diagnostic performance and methodological characteristics of included studies.

Study	Model(s)	Metric	Performance
A. Diagnostic accuracy			
Silva et al. (16)	ChatGPT-3.5	Top-1/Top-2/Top-3 accuracy	25%/57%/68%
Maia-Lima et al. (21)	GPT-4 multimodal	Top-1/Top-2 accuracy	81%/96%
Tassoker (17)	ChatGPT-4o; Experts	Top-1 accuracy	GPT-4o: 78%; Experts: 100%
Hassanein et al. (25)	GPT-4o; DeepSeek-V3; Experts	Top-1/Top-3/Top-5 accuracy	GPT-4o: 38/76/84%; DeepSeek-V3: 51/71/83%; Experts: 59/71/86%
Danesh et al. (29)	ChatGPT-3.5/4; o1-preview	First correct diagnosis	GPT-3.5 40%; GPT-4 62%; o1-preview 80%
Rewthamrongsris et al. (27)	GPT-4o; Gemini; Claude; LLaMA	Accuracy (zero-shot vs. prompt-guided)	GPT-4o 67%–69%; Gemini Flash 69.7%–80.5%; Others 55%–65%
Pradhan (20)	ChatGPT-3.5/4/4o; Gemini	Overall accuracy	36%–67% (models); Experts 71%–74%
Yu et al. (28)	GPT-4o; Claude; Chat-Diagrams	Accuracy (trained vs. untrained)	GPT-4o: 59%–77%; Claude 15%–50%; Chat-Diagrams 64%–68%
Tomo et al. (18)	ChatGPT-3.5/4; Experts	Overall accuracy	GPT-3.5: 65%; GPT-4: 80%; Experts: 87%
Suárez et al. (22)	ChatGPT-4o	Image-only accuracy	58%
Diniz-Freitas et al. (23)	ChatGPT-4V	Accuracy by modality	Text 52%–81%; Image 29%–33%; Multimodal up to 86%
Diniz-Freitas et al. (19)	GPT-4o; DeepSeek-R1	Overall accuracy	GPT-4o: 89%; DeepSeek-R1: 92%; Human baseline: 39%
Cuevas-Nuñez et al. (30)	ChatGPT-4	Overall accuracy	60%
AlFarabi Ali et al. (26)	ChatGPT-4; Copilot; Experts	FDx/DDx	GPT-4: 70%/74%; Copilot: 46%/60%; Experts: 80%/60%
Schmidl et al. (24)	ChatGPT-4	Accuracy by modality	Image: 27%–87%; Text: 20%–80%; Multimodal: 73%–93%
B. Agreement metrics			
Tomo et al. (18)	ChatGPT-3.5/4; Experts	Cohen's κ	Primary diagnosis: 0.532 (GPT-3.5), 0.533 (GPT-4); Alternative diagnosis: 0.337 (GPT-3.5), 0.367 (GPT-4)
Suárez et al. (22)	ChatGPT-4o	Gwet's AC; Percent agreement (Diagnosis)	AC 0.834; PA 0.922
C. Subjective/Perception-Based Outcomes			
Kaygisiz et al. (31)	GPT-4; DeepSeek-V3	Plausibility (Likert)	GPT-4: 3.1; DeepSeek-V3: 4.0

FDx, final diagnosis; DDx, differential diagnosis; Multi, multimodal. Outcomes are grouped by domain. Sections A and B report objective diagnostic performance and reliability metrics related to diagnostic validity relative to the reference standard. Section C reports subjective and perception-based evaluations of output quality and usability, which do not represent diagnostic accuracy or clinical validity*.*

### Diagnostic accuracy

Diagnostic accuracy varied substantially across models, input modalities, and prompting strategies (Table 3). Early-generation models such as ChatGPT-3.5 demonstrated modest Top-1 performance (25%–65%), whereas newer models showed variable accuracy ranging from 36%–89% across different studies and lesion types, with ChatGPT-4 and ChatGPT-4o achieving peak performance of 78%–89% in some clinical datasets, compared with 87%–100% for expert reference standards (16–20).

Multimodal input was associated with the largest performance gains. GPT-4 multimodal achieved Top-1 and Top-2 accuracies of 81% and 96%, while multimodal implementations of ChatGPT-4 and ChatGPT-4 V reached peak accuracies of 86%–93%, exceeding performance observed under text-only (20%–81%) and image-only (27%–87%) conditions (21–24).

Top-k analysis further demonstrated improved diagnostic coverage. In heterogeneous oral lesion cohorts, ChatGPT-4o achieved Top-1, Top-3, and Top-5 accuracies of 38%, 76%, and 84%, with comparable performance reported for DeepSeek-V3 (25). Differential diagnosis tasks showed ChatGPT-4 outperforming Microsoft Copilot (FDx/DDx: 70%/74% vs. 46%/60%), while remaining below expert-level final diagnosis performance (26).

Prompt-guided and trained configurations improved accuracy across multiple models, with GPT-4o achieving 67%–69% accuracy and Gemini Flash up to 80.5% under example-guided prompting, and trained GPT-4o achieving 59%–77% accuracy compared with lower performance for Claude (15%–50%) and Chat-Diagrams (64%–68%) (27, 28). Progressive performance gains across model generations were also observed, with correct first diagnosis rates increasing from 40% (ChatGPT-3.5) to 62% (ChatGPT-4) and 80% (o1-preview) (29). Additional classification tasks reported approximately 60% accuracy for ChatGPT-4 (30).

### Agreement metrics

Only a limited number of studies formally evaluated diagnostic output consistency. Tomo et al. reported moderate repeatability for primary diagnostic hypotheses (Cohen's *κ* ≈ 0.53) and fair consistency for alternative diagnostic classifications (18). In a separate multimodal evaluation, Suárez et al. demonstrated substantial diagnostic repeatability for ChatGPT-4o using Gwet's AC (AC = 0.834) across repeated runs (22). Collectively, these findings indicate that structured clinical context improves output stability; however, variability in final diagnostic classification remains evident.

### Subjective/perception-based outcomes

Subjective and perception-based evaluations primarily assessed output plausibility, reasoning coherence, and perceived usability rather than objective diagnostic performance. Single study incorporating expert Likert-scale scoring reported higher perceived plausibility and contextual grounding of diagnostic reasoning when multimodal inputs were used compared with image-only conditions (31). Importantly, these outcomes reflect expert perception of output quality and should not be interpreted as measures of diagnostic accuracy or clinical validity.

### Clinical utility

Clinical utility was primarily described in terms of decision-support functionality rather than autonomous diagnostic use. Multimodal studies reported that LLM outputs assisted with structuring differential diagnoses, summarizing key diagnostic features, and organizing clinical information to support preliminary clinical reasoning and educational workflows (22, 24). Output stability across repeated runs was considered favorable for training and triage-oriented applications (22). Expert plausibility scoring indicated moderate-to-high perceived usefulness of diagnostic reasoning outputs, with higher ratings observed for more contextually grounded responses (31). However, limitations related to prompt sensitivity, reduced performance in complex cases, and lower interpretability under image-only conditions were consistently highlighted (22, 24). Overall, LLMs were positioned as adjunctive tools that may support clinical reasoning but should not replace expert clinical judgment.

### Risk of bias

**Figure 2:**
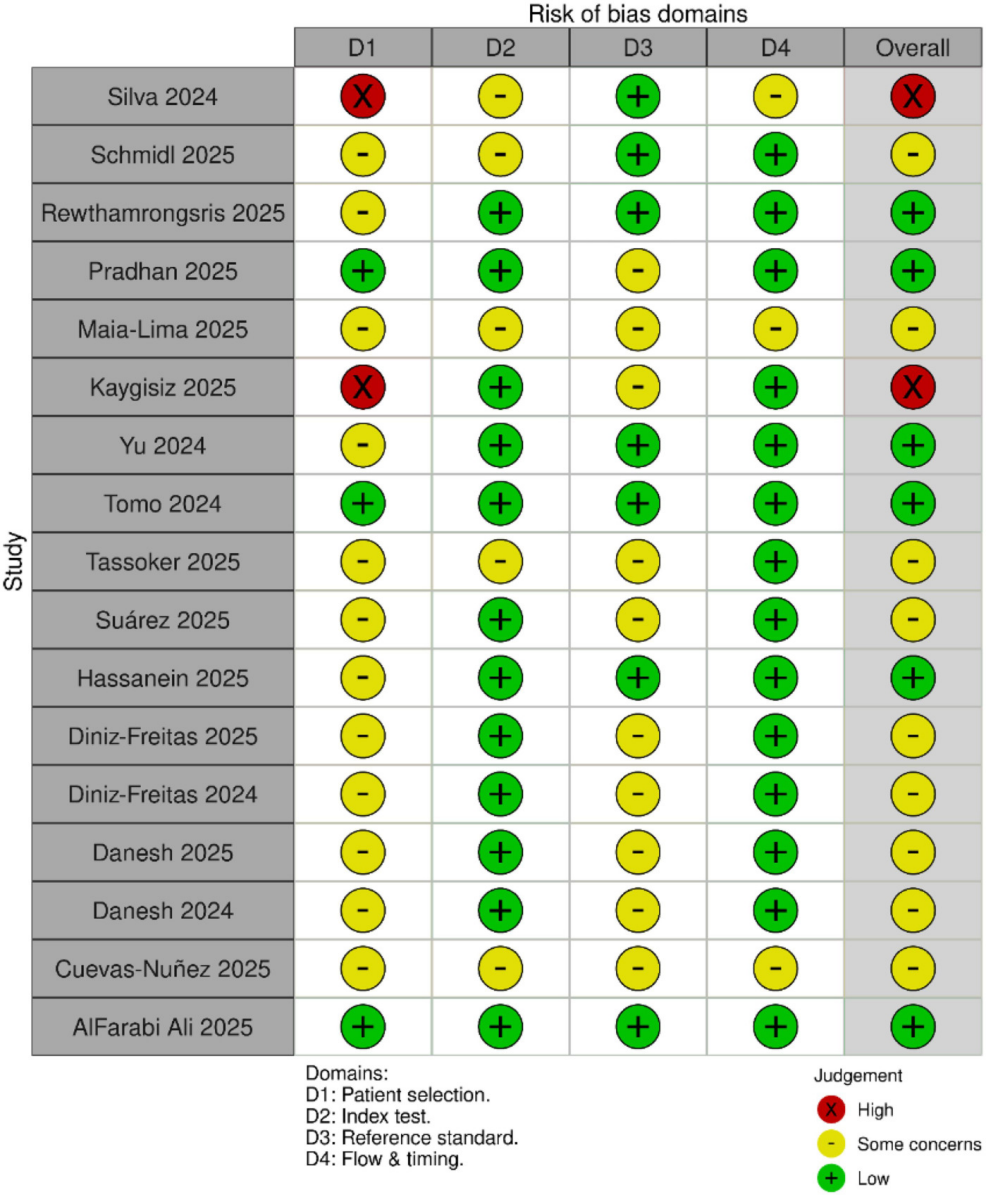
Risk of bias traffic light plot (QUADAS-2) for individual studies.

**Figure 3:**
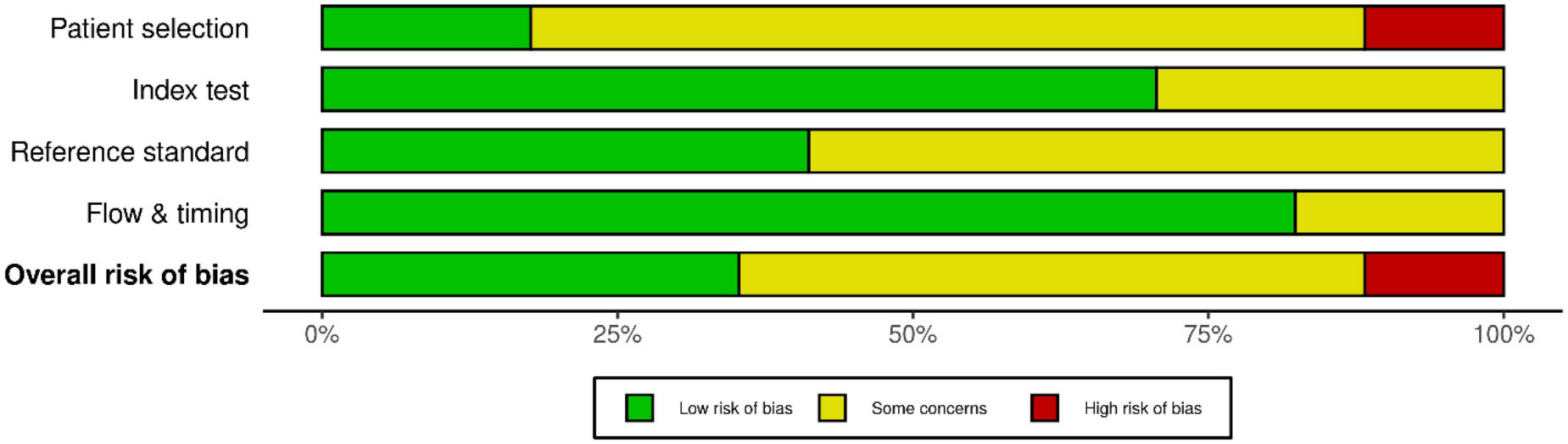
Risk of bias summary (QUADAS-2) across all included studies.

Based on the QUADAS-2 assessment, two studies were rated as high risk of bias, primarily due to non-representative or synthetic data sources. Six studies were rated as low risk, with clear case definitions, appropriate reference standards, and consistent test procedures. The remaining nine studies were judged to have some concerns, most commonly due to convenience sampling, non-uniform reference standards, or incomplete reporting. These findings reflect reasonable but variable methodological quality across the included literature (Figures 2–3).

## Discussion

This systematic review synthesizes recent evidence evaluating large language models (LLMs) for oral lesion diagnosis and identifies three consistent patterns: progressive performance improvement across model generations, substantial gains associated with multimodal input integration, and persistent performance gaps relative to expert clinicians in primary diagnostic accuracy. Although several models demonstrated high benchmark performance under controlled experimental conditions, these findings do not equate to clinical readiness. Instead, current evidence supports the role of LLMs as adjunctive decision-support tools that may enhance clinical reasoning and triage workflows when structured clinical context and imaging data are jointly available, rather than as autonomous diagnostic systems. Accordingly, objective diagnostic performance, reliability metrics, and subjective perception-based outcomes are interpreted separately to avoid conflation of technical performance with clinical utility.

Across included studies, diagnostic performance was strongly influenced by both model architecture and input modality. Earlier-generation models, such as ChatGPT-3.5, consistently demonstrated lower diagnostic accuracy, whereas newer iterations, including ChatGPT-4 and ChatGPT-4o, achieved improved performance, particularly in multimodal settings. However, performance gains were not uniform across tasks, with marked declines observed in image-only diagnostic scenarios. This pattern highlights that architectural improvements alone are insufficient to ensure reliable diagnostic performance and underscores the critical role of clinical context in supporting meaningful reasoning and output stability (32, 33).

A central and reproducible finding across studies was the superiority of multimodal input over unimodal approaches. Combining clinical history with visual data substantially improved diagnostic accuracy, agreement, and perceived output plausibility compared with text-only or image-only inputs. Agreement metrics, including Cohen's κ and Gwet's AC, further supported this trend by demonstrating improved diagnostic repeatability under multimodal conditions. These observations align with emerging evidence that multimodal foundation models more closely approximate human clinical reasoning by integrating visual pattern recognition with contextual symptom interpretation (34). Nevertheless, agreement metrics reflect output consistency rather than diagnostic correctness, and high repeatability should not be interpreted as evidence of clinical validity.

Several studies highlighted the potential clinical value of LLMs as adjunctive diagnostic aids, particularly in settings with limited access to specialist expertise. LLMs demonstrated strengths in structuring differential diagnoses, summarizing discriminative clinical features, and supporting triage-oriented decision-making. These capabilities suggest potential utility in preliminary assessment and educational contexts. However, reasoning depth and diagnostic reliability were often reduced in complex, ambiguous, or atypical cases. Sensitivity to prompt formulation and incomplete contextual interpretation further limit unsupervised deployment. Collectively, these findings reinforce that LLMs may augment clinician reasoning but cannot replace expert judgment.

The present findings extend prior reviews that focused on single-model evaluations or narrow clinical domains. While Panwar and Gupta (2024) examined the diagnostic role of ChatGPT in oral pathology (35) and Liu et al. (2025) focused on dentomaxillofacial radiology applications (34), this review systematically compared multiple LLM families across oral medicine, pathology, radiology, and syndromic diagnostic tasks. This broader scope enabled identification of cross-model performance trends and highlighted multimodal integration as a dominant driver of diagnostic performance, independent of specific model architecture.

Comparison with conventional deep learning approaches further contextualizes the role of LLMs in oral diagnostics. Convolutional neural network–based classifiers have demonstrated high sensitivity and specificity for image-based oral lesion detection (36, 37). However, such models primarily operate on visual pattern recognition. In contrast, LLMs provide complementary capabilities, including explanatory reasoning, clinical feature synthesis, and structured differential diagnosis generation. Rather than competing directly with image classifiers, LLMs may be better positioned as integrative reasoning layers that support broader diagnostic workflows and clinical decision-making.

### Methodological limitations and bias

Despite encouraging trends, the current evidence base is constrained by substantial methodological heterogeneity and threats to validity. Many studies relied on curated benchmark datasets, synthetic case vignettes, or highly selected clinical examples, introducing spectrum bias and case enrichment effects that may inflate apparent diagnostic performance. Reference standards varied widely across studies, ranging from histopathology-confirmed diagnoses to expert consensus and published case solutions, introducing verification bias and subjective variability. Additional heterogeneity in prompting strategies, case presentation formats, lesion categorization schemes, and reported outcome definitions further limited cross-study comparability and precluded quantitative meta-analysis. These findings highlight the urgent need for standardized evaluation frameworks guided by emerging reporting standards such as STARD-AI and QUADAS-AI.

### Real-world applicability and implementation challenges

Translation of experimental performance into real-world clinical deployment remains uncertain. Practical implementation barriers include workflow integration challenges, variable image quality, incomplete clinical histories, regulatory considerations, and the risk of model drift over time. Furthermore, routine oral diagnostic practice involves diagnostic uncertainty, multimorbidity, and atypical lesion presentations that are underrepresented in current evaluation datasets. As such, high benchmark accuracy should not be interpreted as evidence of chairside diagnostic reliability. Prospective, multicenter clinical validation studies will be essential to establish safety, generalizability, and clinical impact.

### Clinical and educational implications

From a clinical and educational perspective, LLMs show promise as adjunctive decision-support tools, particularly in primary care and resource-limited settings where specialist access is constrained. They may assist clinicians in generating differential diagnoses, identifying red flags, and prioritizing further investigations. In dental education, LLMs may support diagnostic reasoning training and case-based learning. However, safeguards are required to mitigate automation bias, hallucination risk, and overreliance on model-generated output.

### Future directions

Future research should prioritize prospective multicenter validation, blinded reader comparison studies, standardized benchmarking protocols, and the development of native multimodal architectures capable of jointly processing clinical text, photographs, radiographs, and histopathology. Additional emphasis should be placed on explainability, bias mitigation, and continuous external validation frameworks to support safe clinical translation.

## Conclusions

Large language models represent a rapidly evolving class of decision-support tools with emerging potential in oral lesion assessment, particularly when multimodal clinical and visual inputs are available. However, current evidence remains heterogeneous, highly context dependent, and methodologically limited by non-uniform reference standards, curated datasets, and variable evaluation designs. Across most head-to-head comparisons, LLMs do not consistently match expert performance in primary diagnostic accuracy and should not be considered autonomous diagnostic systems at this stage. Accordingly, their current role is best positioned as adjunctive tools to support clinical reasoning, triage, and educational applications rather than as replacements for expert judgment. Continued methodological standardization, prospective validation, and real-world clinical evaluation are essential to define their future clinical utility.

## Data Availability

The original contributions presented in the study are included in the article/Supplementary Material, further inquiries can be directed to the corresponding authors.
