# Supplementary material for: Multimodal large language models for oral lesion diagnosis: a systematic review of diagnostic performance and clinical utility
Source: Front Oral Health. 2026 Feb 24;7:1748450. doi: 10.3389/froh.2026.1748450 (PMC12971682; doi:10.3389/froh.2026.1748450)
Supplement: Supplementary file 1 [file Table1.docx]

**Supplementary Appendix 1. Database-specific search strategies (PRISMA-S)**

| **Database** | **Search strategy (verbatim)** | **Hits retrieved** | **Date searched** |
| --- | --- | --- | --- |
| **PubMed (via NLM)** | (("Oral lesions") OR ("Oral cancer") OR ("Oral diseases") OR ("Oral mucosal lesions") OR ("Oral pathology") OR ("Stomatognathic Diseases"[Mesh])) AND ((LLM) OR ("Large language model") OR ("Artificial Intelligence"[Mesh]) OR (ChatGPT) OR (DeepSeek) OR (Gemini) OR (Copilot) OR (Claude) OR (Bard) OR (Grok) OR (AI) OR ("Artificial intelligence")) AND (("Diagnostic accuracy") OR ("Diagnosis") OR ("Differential diagnosis") OR ("Diagnostic Performance") OR ("Diagnosis, Differential"[Mesh])) | 476 | 20 July 2025 |
| **CINAHL (via EBSCO)** | oral lesions AND (artificial intelligence OR ai OR a.i. OR chatbots) AND (diagnostic accuracy OR validity OR reliability OR sensitivity OR specificity) | 172 | 20 July 2025 |
| **Embase (via Elsevier)** | ‘Artificial intelligence’ AND ‘Oral lesions’ | 50 | 20 July 2025 |
| **Web of Science (Core Collection)** | (Oral disease [All Fields]) AND (Large language models [All Fields] OR Chatbots [All Fields]) AND (Diagnosis [All Fields]) | 380 | 20 July 2025 |
| **Google Scholar** | ("Oral lesions") AND (Artificial intelligence) AND (Diagnostic accuracy) | 100 | 20 July 2025 |
