# Supplementary material for: Multimodal large language models for oral lesion diagnosis: a systematic review of diagnostic performance and clinical utility
Source: Front Oral Health. 2026 Feb 24;7:1748450. doi: 10.3389/froh.2026.1748450 (PMC12971682; doi:10.3389/froh.2026.1748450)
Supplement: Supplementary file 2 [file Table2.docx]

**Supplementary Appendix 2. Adapted QUADAS-2 Signaling Questions for LLM Diagnostic Accuracy Studies**

| **Domain** | **Adaptation for LLM/AI Studies** | **Signaling Questions** |
| --- | --- | --- |
| **Case / Vignette Selection** | Refers to how clinical cases, images, or vignettes were sourced for model testing. | 1. Were cases/vignettes selected consecutively or randomly, avoiding cherry-picking? 2. Were inclusion and exclusion criteria clearly described? 3. Was the case spectrum representative of the target clinical population (variety of lesion types, difficulty levels)? |
| **Index Test**  **(LLM Evaluation)** | Refers to the model being evaluated (ChatGPT, Gemini, DeepSeek, etc.), including prompting and blinding. | 1. Was the LLM version explicitly stated? 2. Was the prompting strategy (zero-shot, few-shot, structured) clearly reported and standardized? 3. Were evaluators blinded to the reference standard when judging model output? 4. Was the LLM output assessed without access to training/test leakage? |
| **Reference Standard** | Refers to how the “ground truth” diagnosis was established. | 1. Was the reference standard appropriate (e.g., histopathology, expert consensus, validated dataset)? 2. Was the reference standard applied consistently across all cases? 3. Was the reference independent from the LLM output (avoiding incorporation bias)? |
| **Flow and Timing** | Refers to completeness and consistency of case handling during evaluation. | 1. Were all eligible cases included in the analysis (no selective exclusion)? 2. Were missing or indeterminate LLM outputs handled and reported appropriately? 3. Was the evaluation process consistent across modalities (text, image, multimodal)? |
